# Benefits of Extra Begging Fail to Compensate for Immunological Costs in Southern Shrike (*Lanius meridionalis*) Nestlings

**DOI:** 10.1371/journal.pone.0044647

**Published:** 2012-09-05

**Authors:** Gregorio Moreno-Rueda, Tomás Redondo

**Affiliations:** 1 Departamento de Zoología, Universidad de Granada, Granada, Spain; 2 Estación Biológica de Doñana, Consejo Superior de Investigaciones Científicas, Sevilla, Spain; Dalhousie University, Canada

## Abstract

Theoretical models aimed at explaining the evolution of honest, informative begging signals employed by nestling birds to solicit food from their parents, require that dishonest signalers incur a net viability cost in order to prevent runaway escalation of signal intensity over evolutionary time. Previous attempts to determine such a cost empirically have identified two candidate physiological costs associated with exaggerated begging: a growth and an immunological cost. However, they failed to take into account the fact that those costs are potentially offset by the fact that nestlings that invest more in begging are also likely to obtain more food. In this study, we test experimentally whether a 25% increase in ingested food compensates for growth and immunological costs of extra begging in southern shrike (*Lanius meridionalis*) nestlings. Three nestmates matched by size were given three treatments: low begging, high begging-same food intake, and high begging-extra food intake. We found that, while a higher food intake did effectively compensate for the growth cost, it failed to compensate for the immunological cost, measured as T-cell mediated immune response against an innocuous mitogen. Thus, we show for the first time that escalated begging has an associated physiological net cost likely to affect nestling survival negatively.

## Introduction

Nestling birds typically solicit food from their parents by a set of exuberant begging displays which appear to be excessively complex and wasteful to merely accomplish an efficient transfer of food from parents to young [1], [2]. Ever since Trivers [3], conspicuous begging has been often interpreted as the evolutionary outcome of a genetic conflict of interests between parents and their offspring about the amount of transferred parental resources (Parent-Offspring Conflict), in which offspring are selected to secure more resources from their parents than the latter are selected to give [4]–[6]. From this perspective, showy begging signals may have evolved either as selfish attempts to influence parental decisions in scramble sibling competition for parental resources [7], [8], and/or as honest signals allowing parents to allocate food in proportion to begging intensity, as begging would be a reliable indicator of nestling nutritional need [9]. In both cases, signals that are too cheap to produce should lead to runaway escalation of begging intensity over evolutionary time (as long as more intensive signals are preferred by parents), which might eventually render the communicative system unstable [9], [10]. Most theoretical models for the evolution of honest, information-rich begging signals conclude that a cost function that increases with increasing begging intensity and penalizes misrepresentation is essential for stability [11]–[13].

Escalated begging may incur direct costs (those directly affecting offspring viability or fertility; e.g. reduced growth or immunocompetence, increased vulnerability to predators), as well as indirect costs (those affecting offspring fitness indirectly throughout inclusive fitness, as long as escalated begging is likely to affect the viability and fertility of genetic relatives) [14]. For example, nestling calling and jostling may attract eavesdropping predators to the nest [15]. However, predators typically kill all nestlings in a brood (not only escalating cheaters) and parents may reduce nest vulnerability irrespective of begging [16], hence it is unclear whether predation costs could stabilize honest, informative begging in multi-chick broods [11], [17]. In addition, vigorous posturing, calling and attentiveness by nestlings may incur individual, physiological costs directly proportional to the duration and intensity of begging signals [18], [19]. Muscular and neural activity during begging may increase metabolic expenditure. However, measurements of energetic expenditure during begging episodes suggest it is relatively low [20], though it could affect a nestling energetic budget given the limited scope of developing nestlings to allocate resources to growth (13–28% of total metabolized energy). This may result in a disproportionate decrease in chick viability [21], as long as growth rate may strongly influence juvenile survival [22]. Several studies have found that chicks experimentally induced to beg at higher rates showed reduced growth rates compared to less-begging controls in some species (canaries, *Serinus canaria* [23]; magpies, *Pica pica* [17]; southern shrikes, *Lanius meridionalis* [24]; range of effect size found in these studies, Cohen's *d* = 0.81–0.98) but not others (house sparrow, *Passer domesticus* [25], [26]; ring dove, *Streptopelia risoria* [17]; tree swallow, *Tachicyneta bicolor* [27]; range of effect size, Cohen's *d* = 0.06−0.19). Recently, it has been shown that nestlings begging at high rates incur physiological begging costs in the form of reduced immunocompetence, compared with control nestlings, in house sparrows [26], southern shrikes [24], and magpies [28]. In spite of this evidence, the question of whether begging signals are really costly in terms of offspring fitness still remains a troubling area of disparity between theoretical and empirical studies [14], [29], [30].

If it is the signal cost that maintains honesty, then the marginal cost and marginal benefits coming from signaling have to be equal at equilibrium [31], [32]. An empirical demonstration that direct costs actually stabilize honest, informative begging signals would require to show that (i) nestlings experimentally manipulated into giving exaggerated, out-of-equilibrium signals would suffer a decrease in viability and (ii) that fitness returns (e.g. extra food) accrued by offspring begging at escalated levels should not compensate for the increased costs [33]. Most previous studies have addressed (i) by measuring how experimentally enforced begging levels affect a nestling trait likely to affect viability without manipulating food intake [17], [23], [24], [26], but none of them has so far addressed (ii). A recent field study [34] attempted to quantify the benefits and costs of escalated begging in magpies. In this study, nestlings were given a drug (cyproheptadine), which increases hunger in domestic fowl, pigeons and mammals [35], [36], as a way of increasing begging intensity. Experimental chicks were more likely to gape and receive food from parents, grew up to a better body condition and showed an enhanced immune response at the end of the nestling period. At first sight, these results seem to suggest that benefits of exaggerated begging offset its costs. However, the experimental treatment failed to exert any effect upon time spent begging and postural intensity (the signal attributes likely to increase costs), which casts doubt on its main conclusion that net physiological costs of escalated begging are negligible.

In this study, we analyze whether additional food compensates for exaggerated begging costs (reduced growth and immune response) in southern shrike nestlings. Previously, we found that southern shrike nestlings with exaggerated begging show begging costs in the way of reduced growth and immunocompetence [24]. Here, we created three groups of nestlings matched by nest, age and body mass. The first group begged at a low level and received a standard food amount (low begging-normal food, LB-NF), while a second group was fed the same but forced to beg at a higher level (HB-NF). A third group was also forced to beg intensively, as HB-NF nestlings, but received approximately 25% more food (high begging, extra food, HB-EF). We predicted that, if begging signals are stabilized by growth and immune costs, the extra food received by HB-EF nestlings would not compensate for the costs associated with high begging.

## Materials and Methods

The study was carried out during the spring of 2011, with a population of southern shrikes located at Lomas de Padul (SE Spain). The study area is formed by a mix of shrubland and farmland with scattered Holm oaks (*Quercus ilex*) and kermes oak (*Q. coccifera*) in which most nests were located. Clutches were inspected regularly to determine the exact date of hatching (day 0). The experiment was performed with 24 chicks from 8 nests when nestlings were growing at the highest rate (7-days [37]). In the afternoon of the day before the experiment, we collected one trio of nestlings matched by similar body mass per nest, leaving at least two nestlings to avoid parental desertion. Nestlings were placed in a warm chamber and taken to a laboratory at the Animal Nutrition Unit (Estación Experimental del Zaidin, CSIC). Transport lasted about 30 min. On that afternoon, nestlings were fed *ad libitum*. The day after the experiment, nestlings were fed *ad libitum* again and returned back to their nests during the morning. On the following days, we regularly checked nests to monitor the fate of nestlings used in the experiments; 92% of nestlings tested in the laboratory fledged successfully.

During the experiment, we randomly assigned one nestling from each trio of nestmates to either a treatment: low begging and normal food (LB-NF), high begging and normal food (HB-NF) and high begging and extra food (HB-EF). Nestlings were kept isolated in artificial nests (a ceramic cup lined with clothes), at an ambient temperature of about 36°C. While resting, nestlings were covered by a duster, simulating brooding by the mother. This procedure precluded nestling begging between trials. The experiment started at 8:00 (local hour) and ended at 21:00. Previously, nestlings were weighed with a digital balance (Sartorius; accuracy 0.01 g). We estimated the food to be ingested by nestlings according to their mass during the experimental day, following the allometric relationship calculated by [38]: daily food to be consumed = 0.98×M^0.814^, where M is nestling body mass in grams. For nestlings receiving normal food (LB-NF and HB-NF), we provided approximately 90% of estimated food, while for nestlings receiving extra food (HB-EF), we provided 110% of estimated food. Consequently, HB-EF nestlings received ca. 25% more food than LN-NF and HB-NF nestlings (see Results). Daily food intake was divided in 14 equal portions corresponding to the 14 begging trials; any deviations from expected food intake during an hour were compensated for in subsequent trials. During 2009, we recorded parental feeding rates at 10 nests in the same study area when nestlings were 7-days old, to estimate natural begging and feeding rates by shrike parents. At 6 out of 10 nests, inter-feeding interval per nestling was approximately 1 hour (45–75 min); it was about 30 minutes in three nests, and of 2 hours in the remaining one. Consequently, we established an hourly feeding frequency for experimental trials as it was close to the modal feeding rate in our study population. Food consisted in whole, homogenized boiled chicken egg moistened with water. In each feeding event, food of known mass was given to begging nestlings with forceps. Food was consistently accepted by nestlings. We chose this diet to ensure that all nestlings were receiving a high-quality diet rich in sulphur amino acids [39]. Sulphur amino acids content in the diet is likely to affect positively growth and immune performance [40]–[42].

During each feeding trial, nestlings were stimulated to beg by using acoustic (a characteristic and standardized whistle) and tactile (gently touching their gapes with a forceps) stimuli. However, while LB-NF nestlings were fed immediately after their first gape, HB-NF and HB-EF nestlings were stimulated to beg for 1 min before being fed, holding begging for an average of approximately 30 seconds (Table 1). Therefore, HB-NF and HB-EF nestlings begged for longer than LB-NF nestlings (see Results). All begging trials were recorded with a digital video camera Handycam HDR-XR155E (Sony). From video recordings, we measured (continuous focal sampling, the observer being blind for the treatment) the time each nestling spent begging by using the JWatcher 1.0 software [43]. Two behavioral categories of postural intensity were distinguished: low-intensity begging (gaping, tarsi flexed) and high intensity begging (gaping on extended tarsi, sometimes accompanied by wing flapping), which were assigned ranks 1 and 2, respectively, to establish an average measure of begging postural intensity. The final body mass of nestlings was measured on the next day, at 8:00 h, exactly 24 hours after the first measurement. Mass gain during the experimental day was estimated as final body mass minus initial body mass.

**Table 1:** Mean ± SE values measured for each variable for low begging-normal food (LB-NF), high begging-normal food (HB-NF) and high begging-extra food (HB-EF) nestlings.

	LB-NF (*n* = 8)	HB-NF (*n* = 8)	HB-EF (*n* = 8)
Initial body mass (g)	21.84±1.43	20.30±1.21	21.17±1.26
Food ingested (g)	8.91±0.79	8.10±0.66	10.51±0.85
Time spent begging (s/h)	2.14±0.24	30.34±2.71	28.00±2.69
Mean begging intensity	1.08±0.04	1.12±0.05	1.10±0.04
Growth rate (g)	3.43±0.49	3.23±0.62	4.79±0.70
Immune response (mm)	1.13±0.10	0.73±0.07	0.77±0.06

We also measured how the experimental treatment affected immune response. Immediately before the onset of the experiment, we injected into the left patagium of each chick 0.2 mg of phytohaemagglutinin (PHA-P, L-8754, Sigma Aldrich) diluted in 0.04 ml of isotonic phosphate buffer [44]. PHA-P is an innocuous protein that provokes a T-cell mediated immune response in birds [45], [46], although other components of the immune system are also involved in the response [47]. Previously, we had measured (three times) the patagium thickness with a pressure-sensitive micrometer (Mitutoyo; accuracy: 0.01 mm). At the end of the experiment (24 h later), we again measured the patagium thickness (the measurer being blind for the treatment), calculating the T-cell mediated immune response as the difference between the second and first measurements. The repeatability of measurement was 0.98 (*n* = 8; [48]).

For statistical analyses, we performed General Linear Models (GLM) of Ordinal Least Squares (OLS) with Treatment (fixed factor) as a categorical predictor. In each model, nest of origin was introduced as a random factor to control for variance among nests, thus avoiding pseudoreplication [49]. For every model, we checked for homoscedasticity, and we log-transformed the variable “time begging” in order to fulfil homoscedasticity requirements for statistical analyses. We also checked for normality of residuals, which never deviated from a normal distribution according to a Kolmogorov-Smirnov test (always *p*>0.20; [50]). Means are given with the standard error (SE). All analyses were performed with R 2.15 [51].

### Ethical Note

Based on previous work [24], southern shrike nestlings appeared ideally suited as experimental subjects for this study. The experimental procedure was approved by the CSIC Bioethics Committee (ref. 11_16) and the Ethical Committee for Animal Experimentation at the Animal Nutrition Unit (Estación Experimental del Zaidín, CSIC). In Spain, the southern shrike has been included in the Red List as a result of population decline over the last decades [52]. The study was licensed by the Andalusian authority for wildlife protection (DGGMN-ref SGYB/FOA/AFR 13/01/2011). Following its recommendations, we took any possible measure to minimize disturbance to birds, monitored possible effects of lab procedures on subsequent chick survival and kept sample sizes to the minimum necessary to render statistically meaningful results.

## Results

There were no differences in initial body mass of nestlings according to experimental treatment (*F*_2, 14_ = 0.97, *p* = 0.40; Table 1). Chicks in the "high begging-extra food" (HB-EF) group received 18.1 and 29.8% more food than chicks in the "low begging-normal food" (LB-NF) and "high begging-normal food" (HB-NF) groups, respectively (*F*_2, 14_ = 8.46, *p* = 0.0039; LSD Fisher post hoc, *p*<0.02 in both cases; Table 1), but there were no significant differences between the amount of food ingested by nestlings in the two HB groups (post hoc, *p* = 0.19). HB-NF and HB-EF nestlings, which were stimulated to beg for a longer time, begged for food significantly longer than their LB-NF nestmates (*F*_2, 14_ = 358.77, *p*<0.001; post hoc test, *p*<0.001 in both cases; Table 1). However, there were no significant differences in the time spent begging between HB-EF and HB-NF treatments (*p* = 0.40). Average postural intensity did not differ between the three groups of nestlings (*F*_2, 14_ = 0.19, *p* = 0.83; Table 1). Therefore, the experimental treatment was successful at creating three groups of nestlings, namely LB-NF (low begging, less food), HB-NF (high begging, less food) and HB-EF (high begging, similar to HB-NF nestlings, but eating 29.8% more food).

**Figure 1:**
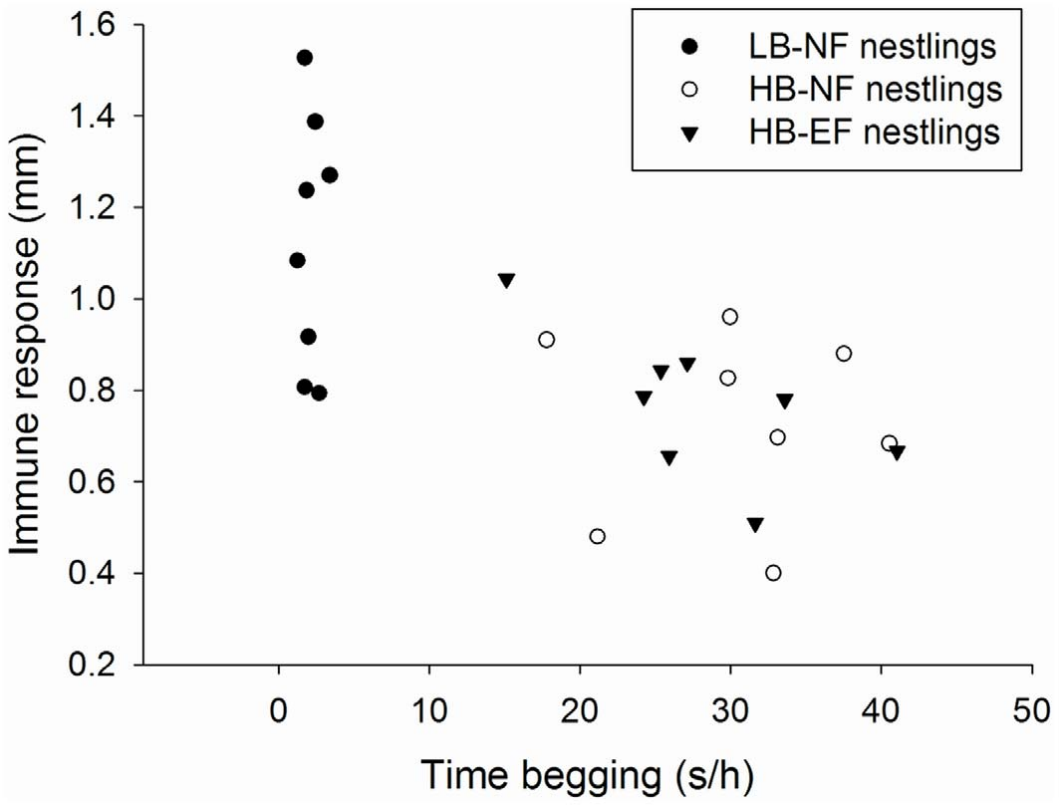
The relationship between immune response to phytohaemagglutinin (patagium thickness in mm) and time begging (in seconds per hour).

We failed to detect a significant effect of begging treatment upon nestling mass gain in this study. HB-EF nestlings, which received more food, gained more mass than HB-NF and LB-NF nestlings (*F*_2, 14_ = 9.51, *p* = 0.002; post hoc, *p*<0.005 in both cases; Table 1). However, HB-NF nestlings mass gain was similar to that in LB-NF nestmates (*p* = 0.61), despite the latter begging for much longer. In contrast, the amount of time begging had a significant effect upon T-cell mediated immune response. Nestlings begging for longer in both the HB-EF and HB-NF groups showed a lower immune response than chicks in the LB-NF group, irrespective of food intake (*F*_2, 14_ = 26.85, *p*<0.001; post hoc, *p*<0.001 in both cases; Table 1). Differences in immune response between HB-NF and HB-EF nestlings were not significant (*p* = 0.53). For the whole sample of chicks, there was a negative correlation between the intensity of the immune response and the amount of time begging which was independent from the amount of food ingested (*â* = −0.66; *F*_1, 14_ = 52.65, *p*<0.001; effect of food received: *F*_1, 14_ = 0.26, *p* = 0.62; Fig. 1).

## Discussion

The experimental protocol induced a measurable negative effect of intensive begging upon immune response irrespective of food intake, but failed to detect a comparable effect upon mass gain by nestling: LB-NF and HB-NF nestlings' mass gain was similar, despite remarkable differences in begging effort. This last result seems at odds with a previous experiment where similar differences in begging times induced by an identical experimental protocol caused a reduction in mass gain in the HB-NF group [24]. In fact, the difference in mass gain between LB and HB nestlings within pairs did not significantly differ between studies ( [24]: 0.86± S.E. = 1.42 g; this study: 0.21±0.74 g; *t*_25_ = 1.23, *p* = 0.23). The main difference between both studies was the type of food received by nestlings: moistened puppy chow (ca. 60% of crude protein content) in [24] and whole boiled egg in this study. HB-NF nestlings fed whole boiled egg gained more daily mass than HB-NF nestlings fed puppy chow in a previous year (3.23±0.62 vs. −0.57±0.27 g; *t*-test, *t*_25_ = 6.62, *p*<0.001), despite ingesting lower amounts of food (8.10±0.66 vs. 10.78±0.52 g; *t*_25_ = −2.94, *p* = 0.007). Similar results were obtained when comparing nestlings in the LB-NF treatment between both years: egg-fed chicks gained more mass (3.43±0.49 g) than puppy chow-fed nestlings (0.29±0.15 g; *t*_25_ = 7.09, *p*<0.001), despite a lower food intake by the former (8.91±0.79 vs. 11.28±0.39 g; *t*_25_ = −3.02, *p* = 0.006). Egg-fed nestlings gained mass at rates similar to those observed in the wild [37]. These results suggest that egg food was of a higher quality, or better assimilated, than puppy chow food. The fact that nestlings begging intensively tended to incur a growth cost only when fed on a lower-quality diet suggests that such a cost may be dependent upon the amount and/or quality of the food received. However, specifically designed experiments are needed to test this idea.

Consistent with our previous findings [24], we found evidence of a reduction in PHA-induced immune response among nestlings begging at a high level, irrespective of food intake. Immune response (i.e. patagium swelling) values were higher in this than in the 2011 study (HB nestlings: 0.73±0.07 vs. 0.34±0.03 mm; LB nestlings: 1.13±0.10 vs. 0.44±0.05 mm; *t*_25_≥6.00, *p*<0.001 in both cases), which may again be indicative of a positive effect of diet quality upon immune response. It is known that both protein and sulphur amino acids-rich diets improve immune function [40], [41]. However, despite being fed such a high quality food, those nestlings that begged for longer mounted a weaker immune response than their less begging nestmates, and such an immunological cost was not compensated for by a higher food intake. Chicks that begged for longer and ate more food (HB-EF) showed an immune response 31.9% lower than nestlings that begged less and ate less food (LB-NF). This result contrasts with the findings of a study [34] in which magpie nestlings treated with cyproheptadine, an appetite enhancer, received more food and mounted a higher immune response at the end of the nestling period. In that study, however, the experimental treatment failed to induce differences in total time begging or postural intensity between nestlings and therefore the authors’ assumption that treatment affected signal intensity (hence costs) seems unwarranted. Moreover, experimental chicks were (for unknown reasons) more efficient at obtaining food than their control nestmates, which implies that, in fact, they may have incurred lower net costs. On the other hand, their study lasted for various days in magpie nestlings development (our study only lasted for 24 h), and therefore it is possible that the compensatory effect of extra food is not apparent within 24 h. However, extra resources probably are assimilated by nestlings within 24 h and used in immune system, as evidenced by the fact that nestlings in this study, fed with richer food, showed higher immune response than in our previous study [24], in which they were fed with a poorer food (see above).

An offspring’s optimal begging level is determined by the Benefit/Cost balance which maximizes its inclusive fitness [9]. When a nestling is experimentally forced to beg at out-of-optimal levels, the B/C relationship becomes altered and a lower fitness gain is expected. Begging rates induced in this study (about 2–35 s/hour) were well below the average recorded under natural field conditions (99.9±19.0 s/h; [24]) and, consequently, they are within the strategic range that nestlings may choose to display in the wild. Our results show that nestlings begging for food more intensively may accrue benefits in the form of an enhanced mass gain as long as parents provide them with more food (e.g. [53]; but see [54]). At this point, note that we arbitrarily chose an increase of 25% of food, but it is unknown whether parents in the field would respond to exaggerated begging by an increase in food supply similar, higher, or smaller than this. However, nestlings begging more also incur a cost in the form of reduced immunocompetence which is not compensated for by extra feeding. Optimal begging levels will result from the interplay between maintaining an adequate growth rate without compromising immune response, as well as other physiological traits (e.g., oxidative stress [28]). The B/C relationship depends on benefits as well as costs of begging in terms of fitness (survival and breeding success) for both a focal nestling and its parents and siblings (indirect costs and benefits). In any sense, our results raise the question of whether fitness benefits accrued via an enhanced growth rate are high enough to compensate for immunological costs, a balance which is likely to be affected by many ecological factors (i.e., pathogen prevalence, mass-dependent juvenile survival, etc.) likely to vary among different species and even populations. However, our results fit remarkably well with the basic assumptions of signaling models (e.g. [55]), namely that signal intensity at equilibrium is determined by the balance between a benefit function which enhances nestling survival in direct proportion to begging level, but which is comparatively lower for chicks in good nutritional condition (i.e. a well-fed chick will accrue a lower marginal growth gain than a needy chick for the same amount of food) and a cost function which monotonically decreases chick survival in proportion to begging intensity but which is independent from nestling condition. This study, by successfully manipulating the benefit/cost ratio of begging behavior for the first time, suggests that benefits and costs may be mediated by different proxies of fitness that must be taken into account in future studies.
